# Higher C-Reactive Protein to Albumin Ratio Portends Long-Term Mortality in Patients with Chronic Heart Failure and Reduced Ejection Fraction

**DOI:** 10.3390/medicina60030441

**Published:** 2024-03-07

**Authors:** Veysel Ozan Tanık, Evliya Akdeniz, Tufan Çınar, Barış Şimşek, Duygu İnan, Ahmet Kıvrak, Yavuz Karabağ, Metin Çağdaş, Kamuran Kalkan, Can Yücel Karabay, Bülent Özlek

**Affiliations:** 1 Department of Cardiology, Etlik City Hospital, Health Sciences University, Ankara 06170, Turkey; 2 Department of Cardiology, School of Medicine, Baskent University, Istanbul 34662, Turkey; 3 Department of Cardiology, Sultan II. Abdülhamid Han Training and Research Hospital, Health Sciences University, Istanbul 34668, Turkey; 4 Department of Cardiology, Dr. Siyami Ersek Thoracic and Cardiovascular Surgery Training and Research Hospital, Health Sciences University, Istanbul 34668, Turkey; 5 Department of Cardiology, Başakşehir Çam Sakura City Hospital, Health Sciences University, Istanbul 34480, Turkey; 6 Department of Cardiology, School of Medicine, Kafkas University, Kars 36000, Turkey; 7 Department of Cardiology, School of Medicine, Mugla Sitki Kocman University, Mugla 48000, Turkey; bulent_ozlek@hotmail.com

**Keywords:** albumin, C-reactive protein, heart failure with reduced ejection fraction, mortality

## Abstract

*Background and Objectives*: In this study, we aimed to investigate the prognostic value of the C-reactive protein to albumin ratio (CAR) for all-cause mortality in patients with chronic heart failure with reduced ejection fraction (HFrEF). *Materials and Methods*: In total, 404 chronic HFrEF patients were included in this observational and retrospective study. The CAR value of each patient included in this analysis was calculated. We stratified the study population into tertiles (T1, T2, and T3) according to CAR values. The primary outcome of the analysis was to determine all-cause mortality. *Results*: The median follow-up period in our study was 30 months. In the follow-up, 162 (40%) patients died. The median value of CAR was higher in patients who did not survive during the follow-up [6.7 (IQR = 1.6–20.4) vs. 0.6 (IQR = 0.1–2.6), *p* < 0.001]. In addition, patients in the T3 tertile (patients with the highest CAR) had a higher rate of all-cause mortality [n = 90 cases (66.2%), *p* < 0.001]. Multivariate Cox regression analysis revealed that CAR was an independent predictor of mortality in patients with HFrEF (hazard ratio: 1.852, 95% confidence interval: 1.124–2.581, *p* = 0.005). In a receiver operating characteristic curve analysis, the optimal cut-off value of CAR was >2.78, with a sensitivity of 66.7% and specificity of 76%. Furthermore, older age, elevated N-terminal pro-brain natriuretic peptide levels, and absence of a cardiac device were also independently associated with all-cause death in HFrEF patients after 2.5 years of follow-up. *Conclusions*: The present study revealed that CAR independently predicts long-term mortality in chronic HFrEF patients. CAR may be used to predict mortality among these patients as a simple and easily obtainable inflammatory marker.

## 1. Introduction

Heart failure with reduced ejection fraction (HFrEF) is a clinical syndrome that usually occurs due to reduced cardiac output or increased intracardiac pressures [1]. Although the temporal trend of HFrEF incidence shows a modest decline in recent years, its impact on the burden of health economics is still significant [2,3]. Neurohormonal activation as a response to impaired systolic function of the heart has been the most well-established mechanism underlying HFrEF progression and left ventricular (LV) remodeling [4]. In addition to neurohormonal activation, circulating levels of proinflammatory cytokines have been the main focus of interest for understanding HFrEF progression. It has been shown that increased serum levels of proinflammatory cytokines, such as tumor necrosis factor-alpha (TNF-α) and interleukin-6 (IL-6), may indicate a worse prognosis and poor functional classification of the New York Heart Association [5,6]. Moreover, serum C-reactive protein (CRP), which is the most widely investigated inflammatory marker, has a prognostic implication in terms of survival in patients with HFrEF [7]. In addition to that, the proinflammatory nature of HFrEF can lead to a decreased serum albumin level, even though its level may vary depending on the clinical status of the patients. Hypoalbuminemia, which was associated with poor survival rates in patients with heart failure (HF), was encountered in approximately 25% of such cases [8].

The C-reactive protein (CRP) to albumin ratio (CAR), recently introduced as a new inflammation index, combines CRP and albumin into a single index. Previous clinical studies have shown that CAR is a better inflammatory index to reflect the prognosis of patients with multiple critical diseases and malignancy than CRP and albumin alone [9,10]. Moreover, this index has been shown to better predict in-hospital and long-term mortality in patients with ST-segment elevation myocardial infarction [11]. Since HFrEF is associated with an elevated inflammatory state, the present study aimed to evaluate the predictive value of CAR for all-cause mortality in patients with chronic HFrEF.

## 2. Materials and Methods

### 2.1. Study Setting and Participants

Firstly, 420 patients with HFrEF were enroled in this observational and retrospective study. The cohort consisted of consecutive adult patients with chronic HFrEF admitted to the cardiology outpatient clinic between January 2015 and March 2015. The HFrEF diagnosis was established per the recommendations of the 2021 European Society of Cardiology HF guidelines [1]. Patients with known evidence of acute myocardial ischemia, cardiogenic shock, acute HF, hematological disease, neoplastic metastases to the bone marrow, sepsis, pregnancy, inflammatory bowel diseases, chronic inflammatory conditions, a history of glucocorticoid three months before admission, and/or other extracellular fluid-retention diseases (e.g., hypothyroidism and liver cirrhosis) were excluded (n = 10 cases, 2.4%). In addition, a total of 6 patients (1.4%) were excluded from the study due to loss of follow-up data. Finally, 404 patients with HFrEF were analyzed in this study.

This study complied with the Declaration of Helsinki, and all study protocols were approved by the institutional review board of Haydarpasa Numune Training and Research Hospital (approval date and number: 8 April 2019–2019/48/814). Informed consent was waived by the Ethics Committee due to the retrospective nature of the study.

### 2.2. Data Collection

Clinic and laboratory data were retrieved from the hospital’s electronic database. The blood samples were collected from all patients to measure the baseline values of albumin, creatinine, glucose, and CRP at hospital admission. Serum creatinine, CRP, and albumin levels were determined using an automatic biochemical analyzer (Roche Diagnostics Cobas 8000 c502, Indianapolis, IN, USA). The normal values for CRP and albumin levels were 0–1 mg/dL and 3.5–5.2 g/dL, respectively. CAR was calculated as the ratio of CRP to albumin level multiplied by 100. LVEF was assessed by transthoracic echocardiography. For measurement of LVEF, the modified biplane Simpson’s rule was performed. 

### 2.3. Follow-Up and Endpoint

The follow-up period for this study concluded in March 2019. The primary outcome of this research was all-cause mortality. To determine the main outcome of the study, either follow-up interviews or visiting outpatient clinics or hospital electronic databases were used. In addition, we used the National Death Registry System to confirm the death of each case included in the study. 

### 2.4. Statistical Analysis

Statistical analyses were performed using SPSS version 22.0 (IBM, Chicago, IL, USA). The Kolmogorov–Smirnov test was used to determine the normality of the data. Continuous variables with normal distribution were expressed as mean ± standard deviation. These parameters were assessed using the analysis of variance test. Variables without normal distribution were expressed as the median and interquartile range (IQR) and were compared using the Kruskal–Wallis H-test. The categorical variables were expressed as numbers (percentages) and were compared using Fisher’s exact test or χ^2^-test. Patients were divided into three groups according to baseline CAR values. During the process of determining the cut-off values that separate the three patient groups, these values were determined to ensure that there was no significant difference in terms of the number of patients between the groups. This approach aimed to maintain fairness and avoid any potential bias or imbalance that could influence the results. The Kaplan–Meier survival curve analysis was performed to determine event-free survival curves according to the CAR tertiles. Both univariate and multivariate Cox proportional hazard analyses were performed to determine independent predictors of mortality. Variables with *p*-value < 0.05 according to univariate analysis were included in multivariate analysis. A receiver operating characteristic (ROC) curve analysis was conducted to determine the optimal cut-off value of CAR in predicting mortality. A *p*-value < 0.05 was considered statistically significant.

## 3. Results

The mean age of the population was 61 ± 13 years, and 124 (30.7%) patients were women. In total, 162 (40%) cases died during the follow-up. The median follow-up was 30 (IQR: 4–40) months. Firstly, the study population was divided into survivor and non-survivor groups (Table 1). Patients in the non-survivor group were more likely to be older (66 vs. 55 years, *p* < 0.001). The prevalence of hypertension, hyperlipidemia, coronary artery disease (CAD), chronic renal failure (CRF), and atrial fibrillation (AF) was higher in patients who died during the follow-up. The other baseline features were not different between the groups. LVEF was slightly higher in survivors than in deceased patients (26 vs. 25%, *p* = 0.022). There were no significant differences in medication usage between both groups. In regard to laboratory findings, patients who died during the follow-up had higher N-terminal pro-brain natriuretic peptide (NT-pro-BNP), troponin I, creatinine, CRP, and CAR (6.7 (IQR = 1.6–20.4) vs. 0.6 mg/dL (IQR = 0.1–2.6), *p* < 0.001) values but lower values of total protein, albumin, hemoglobin, and lymphocyte. On the other hand, deceased patients received less cardiac device therapy, including implantable cardioverter-defibrillator (ICD) or cardiac resynchronization therapy (CRT).

Based on the CAR values, we divided the study population into tertiles as T1, T2, and T3 (Table 2). Patients with CAR value < 0.4 (n = 132 cases) were included in the T1 tertile, patients with CAR value = 0.4–5.9 (n = 136 cases) were included in the T2 tertile, and those with CAR value ≥ 5.9 were included in the T3 tertile (n = 136 cases). Patients stratified into the T3 tertile were older (70 vs. 58 vs. 50 years, respectively; *p* < 0.001), and the frequency of hypertension, diabetes, hyperlipidemia, CRF, CAD, and AF was more common. LVEF was significantly lower in patients stratified into the T3 tertile (*p* < 0.001). No significant difference was found between the three groups regarding drug therapies except that statin and P2Y12-receptor inhibitor use was higher in the T2 and T3 groups. We observed that NT-pro-BNP (7590 vs. 1252.5 vs. 609.5 ng/L, respectively; *p* < 0.001), troponin I, creatinine, CRP, and CAR (0.1 (IQR = 0–0.2) vs 1.6 (IQR = 0.9–2.6) vs 18.7 (IQR = 9.2–28.1), respectively; *p* < 0.001) were higher in the T3 tertile. In contrast, those patients included in the T3 tertile had lower levels of total cholesterol, total protein, albumin, hemoglobin, neutrophil, and lymphocyte. Notably, patients in the group with the highest CAR (T3) had a significantly higher prevalence of cardiac devices than those in the T1 and T2 groups (69.9 vs. 59.6 and 50%, respectively; *p* = 0.004). Furthermore, when comparing the three groups, the group with a higher CAR rate showed a significantly higher all-cause mortality rate compared to the group with a lower CAR rate (66.2 vs. 36.8 and 16.7%, respectively; *p* < 0.001).

Independent predictors of long-term mortality according to univariate and multivariate Cox regression analyses are shown in Table 3. Older age, CAD, hypertension, hyperlipidemia, CRF, AF, decreased LVEF, absence of cardiac devices, creatinine, elevated NT-pro-BNP, troponin I, decreased hemoglobin, and higher CAR were predictors of all-cause mortality based on univariate analysis. After the inclusion of these variables into multivariate Cox regression analyses, we noted that advanced age (hazard ratio (HR): 2.008, 95% confidence interval (CI): 1.753–2.309, *p* = 0.001), the absence of cardiac device therapy (HR: 2.458, 95% CI: 1.592–4.875, *p* = 0.001), elevated NT-pro-BNP levels (HR: 1.967, 95% CI: 1.108–2.709, *p* = 0.002), and higher CAR (HR: 1.852, 95% CI: 1.124–2.581, *p* = 0.005) were independently associated with mortality.

**Figure 1:**
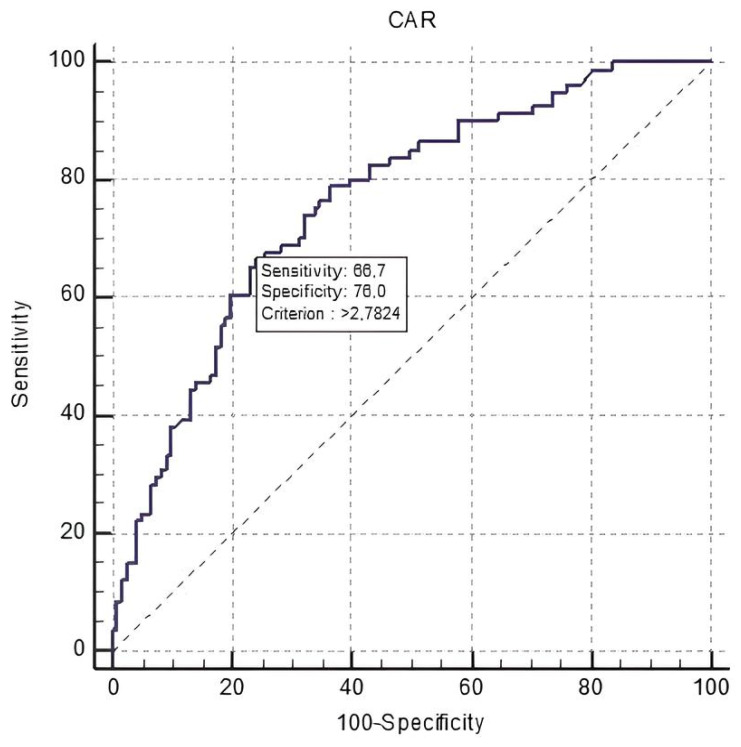
A receiver operating characteristic (ROC) curve analysis of C-reactive protein to albumin ratio (CAR) in predicting long-term mortality.

**Figure 2:**
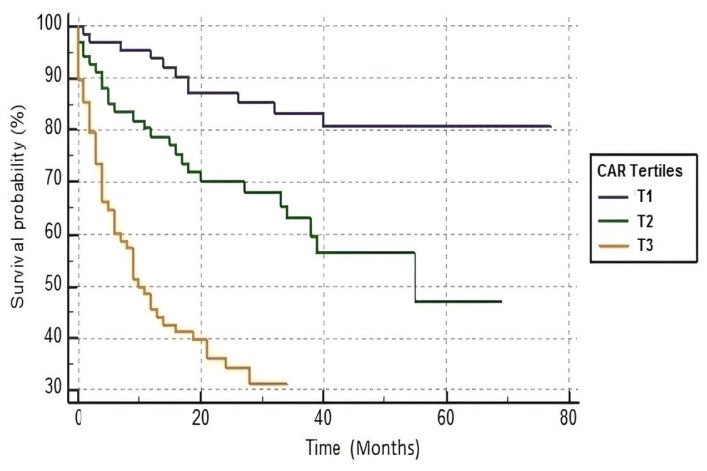
Kaplan–Meier survival analysis of patients according to C-reactive protein to albumin ratio (CAR) tertiles.

An ROC curve analysis demonstrated that CAR level > 2.78 predicted mortality with a sensitivity of 66.7% and specificity of 76% (AUC: 0.762; 95% CI: 0.697–0.819, *p* < 0.001) (Figure 1). The Kaplan–Meier survival analysis showed significant differences between tertiles and survival rates according to CAR values (log-rank *p* = 0.001) (Figure 2).

## 4. Discussion

The main findings of the current study can be summarized as follows: (i) the high admission CAR level had a predictive value for all-cause mortality in patients with chronic HFrEF; (ii) older age, higher NT-pro-BNP levels, and absence of cardiac device therapy were identified as other predictors of mortality; (iii) after controlling for confounders, the cut-off value of CAR for predicting mortality was found to be greater than 2.78, with a sensitivity of 0.66 and specificity of 0.76.

HF causes a severe inflammatory response in the body, resulting in elevated CRP levels and decreased albumin levels [5,6,12]. Proinflammatory cytokines and inflammatory response to myocardial insult may have deleterious effects on contractile function and myocardial remodeling, which might have an impact on clinical status due to worsening HF or death [13,14]. Beyond the prognostic value of CRP in patients with an established diagnosis of HF, it might have the role of predicting HF development in patients without prior myocardial infarction [15]. Furthermore, the BASEL study found that higher plasma CRP levels on admission predicted a higher risk of mortality at two years, as well as readmission in HF patients [14]. The Japanese ATTEND study demonstrated that the value of elevated CRP levels can be used to predict the risk of cardiovascular and non-cardiovascular death six months after discharge in HF patients [16]. In addition, The Korean Heart Failure Registry, which included a large heterogeneous group of HF cases, demonstrated that CRP values on admission could help predict the risk of death 12 months after the index hospitalization, especially when combined with natriuretic peptide levels [17]. On the other hand, few recent studies conducted in acute and chronic HF have shown that albumin predicted in-hospital outcome and survival up to 1 year after measurement [18,19]. Malnutrition, inflammation, and fluid retention can all be cited as factors contributing to the presence of hypoalbuminemia in the course of HF [20]. All these studies mentioned above emphasize that CRP and albumin values have an essential role in determining the prognosis of HF patients. In addition to CRP and albumin, different scoring systems, including variable inflammatory markers (like white blood cell count, procalcitonin, NT-pro-BNP, and troponin) and clinical features (like hypertension, CRF, and AF) were examined to determine acute and long-term negative outcomes in patients with HF [21,22,23,24]. Our study used the CAR value, a newly used marker compared to others, to determine the prognosis in patients with chronic HFrEF. This value was shown to be at least as sensitive as other parameters.

The CAR is a newly defined systemic inflammatory index that combines levels of CRP (positive acute phase reactant) and albumin (negative acute phase reactant) [25]. It is a novel index that can be used to predict adverse clinical outcomes due to its availability, practicability, and low cost. The exact pathophysiological mechanisms of the relationship between CAR and adverse clinical events have not been established well. However, as an acute phase reactant, CRP elevation induced by IL-6 as a response to inflammatory processes was introduced many years ago [26]. Otherwise, CRP has an important role in vascular inflammation and atherosclerotic plaque formation [27]. As inverse to CRP, albumin is a negative acute phase reactant, the plasma level of which shows a decline in case of inflammation [28]. Taken together, these two indicators are combined into a single inflammation-based index as CAR, which had been previously evaluated in patients with malignancy and acute illness, and it was observed that an elevated CAR was closely related to poor prognosis among these patients [9,29,30].

As more studies on this subject have increased in recent years, CAR may find a place in the cardiology practice regarding prognostic inference. The role of CAR in determining the severity of CAD in patients with stable angina pectoris and those with acute coronary syndromes (ACS) has been established in recent years. These studies suggest that CAR significantly predicts CAD severity and complexity in the clinical setting of both stable angina pectoris and ACS [31,32,33]. However, only four studies in the literature have looked into the impact of CAR on the prognosis of chronic HFrEF patients. In one of the most recent retrospective analyses conducted by Çinier et al., HFrEF patients with ICD were monitored for a median of 38 months, and it was found that elevated CAR levels were linked to all-cause mortality [34]. Using CRP and albumin parameters, Cho et al. conducted a study on the predictive significance of the modified Glasgow prognostic score (mGPS) in chronic HFrEF patients. They discovered that a high mGPS value, characterized by elevated CRP and low albumin, predicted all-cause mortality [35]. Similarly, in another retrospective study, a high mGPS score was found to be a prognostic indicator for hospitalization and mortality in 306 chronic HFrEF patients undergoing CRT [36]. Our study’s findings support the results of the three aforementioned studies. In a very small study consisting of a prospective 12-month follow-up of 77 patients with LVEF < 50%, a CRP to albumin ratio of 1.2 and above was associated with increased hospitalizations but had no effect on mortality [37]. However, it is important to note that the number of patients in this study was very small, the follow-up period was short, the CRP albumin ratio cut-off value was meager, and patients with LVEF between 40 and 50% were included in the study, which are all important limitations that may impact the outcomes [37].

Treatment modalities such as CRT and ICD have been proven to enhance survival rates in patients with advanced HFrEF [1]. Our research reveals that the frequency of cardiac devices is higher, particularly in the patient group with the highest CAR. These data reinforce the notion that CAR values are higher in patients with more severe HF. Furthermore, our study indicates that older age and NT-pro-BNP levels, which have been demonstrated as prognostic markers in HF patients in numerous prior studies [38], are linked to elevated CAR levels. These findings provide further evidence of the predictive power of CAR. 

### Study Limitations

This study has some significant limitations. First, the design of the research was retrospective. Second, the study included a limited number of HFrEF cases. Third, even though multivariate analysis was conducted to determine independent predictors of mortality, some unmeasured confounders might be present, which might affect the results of the study. Fourth, spot laboratory data were used to determine the relationship between CAR and all-cause mortality in HF patients. Fifth, we did not collect data regarding cardiac and non-cardiac mortality. Finally, more prospective studies with large sample sizes are needed to confirm the relationship between CAR and mortality in patients with HFrEF.

## 5. Conclusions

As HF is a complex, multisystem, and challenging clinical syndrome, CAR is practical and essential in determining the risk of adverse events in clinical practice. Therefore, we showed that CAR as a novel index can be used to predict long-term adverse outcomes in patients with chronic HFrEF. Given the changes in CRP and albumin plasma concentrations and their prognostic significance in the spectrum of HF syndrome, it is understandable why CAR can predict mortality in our study. CAR is a simple, inexpensive, and readily available marker which may have a role in determining the risk of mortality in patients with stable HFrEF. However, this subject needs to be investigated with further studies with a larger population.

## Figures and Tables

**Table 1:** Baseline demographic characteristics, medications, and laboratory results of survivors and non-survivors.

	Survivor Group (n = 242)	Non-Survivor Group (n = 162)	*p* Value
Age, years	55 ± 13	66 ± 14	<0.001
Female gender, n (%)	74 (30.6)	50 (30.9)	0.966
History			
Hypertension, n (%)	118 (48.8)	116 (71.6)	0.001
Diabetes mellitus, n (%)	92 (38.0)	56 (34.6)	0.618
Hyperlipidemia, n (%)	42 (17.4)	50 (30.9)	0.025
Smoking history, n (%)	134 (55.4)	80 (49.4)	0.403
CRF, n (%)	36 (14.9)	52 (32.1)	0.004
CAD, n (%)	100 (41.3)	98 (60.5)	0.008
CVD, n (%)	16 (6.7)	14 (8.8)	0.584
AF, n (%)	56 (23.1)	74 (45.7)	0.001
At admission			
SBP, mmHg	136 ± 29	138 ± 37	0.072
Heart rate, bpm	87 ± 8	89 ± 10	0.082
LVEF, %	26 (19–33)	25 (22–33)	0.022
Medication			
Beta-blocker, n (%)	240 (99.2)	160 (98.8)	0.774
ACE-i or ARB, n (%)	222 (91.7)	134 (82.7)	0.052
MRA, n (%)	146 (60.3)	88 (54.3)	0.396
P_2_Y_12_ inhibitors, n (%)	32 (13.2)	38 (23.5)	0.060
ASA, n (%)	142 (58.7)	86 (53.1)	0.432
Statin, n (%)	92 (38.0)	68 (42.0)	0.573
Digoxin, n (%)	24 (9.9)	30 (18.5)	0.078
VKA, n (%)	90 (37.2)	78 (48.1)	0.121
NOAC, n (%)	20 (8.3)	20 (12.3)	0.341
Laboratory variables			
Glucose, mg/dL	105 (91–134)	104 (92–140)	0.969
NT-pro-BNP, ng/L	898 (311–1837)	2575 (1150–8770)	<0.001
Troponin I, ng/dL	0.03 (0.01–0.07)	0.05 (0.035–0.12)	0.040
Creatinine, mg/dL	1.05 (0.84–1.3)	1.26 (0.96–1.5)	0.003
Sodium, mEq/L	136.4 ± 4.0	136.6 ± 4.7	0.660
Potassium, mEq/L	4.3 ± 0.6	4.4 ± 0.6	0.146
Magnesium, mg/dL	2.02 ± 0.36	2.08 ± 0.29	0.335
Total cholesterol, mg/dL	160 (138–200)	160 (124–186)	0.118
Total protein, g/dL	6.77 ± 0.78	6.36 ± 0.83	<0.001
Albumin, g/dL	3.80 ± 0.52	3.36 ± 0.58	<0.001
CRP, mg/dL	2.0 (0.4–10.1)	22.3 (6.0–67.0)	<0.001
WBC count, cells/µL	8.9 ± 2.7	9.0 ± 3.1	0.190
Hemoglobin, g/dL	12.7 ± 1.8	11.9 ± 2.0	0.007
Platelet, 10^3^/µL	243 ± 80	223 ± 95	0.113
Neutrophil, 10^3^/µL	5.7 ± 2.5	6.3 ± 3.1	0.014
Lymphocyte, 10^3^/µL	2.1 ± 1.3	1.7 ± 1.2	0.035
CAR, ×100	0.6 (0.1–2.6)	6.7 (1.6–20.4)	<0.001
Cardiac devices (ICD or CRT), n (%)	168 (69.4)	74 (45.7)	<0.001
Follow-up duration, months	34 (24–47)	7 (2–16)	<0.001

Abbreviations: CRF, chronic renal failure; CAD, coronary artery disease; CVD, cerebrovascular disease; AF, atrial fibrillation; SBP, systolic blood pressure; LVEF, left ventricle ejection fraction; ACE-i, angiotensinogen converting enzyme inhibitor; ARB, angiotensinogen receptor blocker; MRA, mineralocorticoid receptor blocker; ASA, acetylsalicylic acid; VKA, vitamin K antagonist; NOAC, novel oral anticoagulant; NT-pro-BNP, N-terminal pro-brain natriuretic peptide; CRP, C-reactive protein; WBC, white blood cell count; CAR, C-reactive protein to albumin ratio; ICD, implantable cardioverter-defibrillator; CRT, cardiac resynchronization therapy.

**Table 2:** Demographic characteristics, laboratory results, and mortality rates of all patients according to CAR values.

	T1 [0 < CAR < 0.4] (n = 132)	T2 [0.4 ≤ CAR < 5.9] (n = 136)	T3 [CAR ≥ 5.9] (n = 136)	*p* Value
Age, years	50 ± 11	58 ± 14	70 ± 11	<0.001
Female gender, n (%)	36 (27.3)	42 (30.9)	46 (33.8)	0.713
History				
Hypertension, n (%)	36 (27.3)	78 (57.4)	120 (88.2)	<0.001
Diabetes mellitus, n (%)	30 (22.7)	56 (41.2)	62 (45.6)	0.015
Hyperlipidemia, n (%)	2 (1.5)	32 (23.5)	58 (42.6)	<0.001
Smoking history, n (%)	60 (45.4)	66 (48.5)	88 (64.7)	0.055
CRF, n (%)	12 (9.1)	24 (17.6)	52 (38.2)	<0.001
CAD, n (%)	34 (25.8)	76 (55.9)	88 (64.7)	<0.001
CVD, n (%)	8 (6.1)	10 (7.6)	12 (8.8)	0.831
AF, n (%)	28 (21.2)	38 (27.9)	64 (47.1)	0.004
At admission				
SBP, mmHg	125 ± 14	128 ± 12	125 ± 10	0.812
Heart rate, bpm	87 ± 8	88 ± 9	88 ± 9	0.508
LVEF, %	33 (26–38)	24 (18–32)	23 (18–29)	<0.001
Medications				
Beta-blocker, n (%)	130 (98.5)	136 (100)	134 (98.5)	0.599
ACE-i or ARB, n (%)	112 (84.8)	120 (88.2)	124 (91.1)	0.526
MRA, n (%)	72 (54.5)	80 (58.8)	82 (60.2)	0.783
P_2_Y_12_ inhibitors, n (%)	10 (7.6)	30 (22.1)	30 (22.1)	0.039
ASA, n (%)	68 (51.5)	78 (57.4)	82 (60.3)	0.581
Statin, n (%)	20 (15.2)	70 (51.5)	70 (51.5)	<0.001
Digoxin, n (%)	10 (7.6)	16 (11.8)	28 (20.6)	0.077
VKA, n (%)	52 (39.4)	64 (47.1)	52 (38.2)	0.526
NOAC, n (%)	12 (9.1)	14 (10.3)	14 (10.3)	0.965
Laboratory results				
Glucose, mg/dL	101 (89–128)	105 (93–131)	119 (93–149)	0.230
NT-pro-BNP, ng/L	609.5 (221–1614)	1252.5 (552–2575)	7590 (2950–11,600)	<0.001
Troponin I, ng/dL	0.025 (0.01–0.08)	0.04 (0.02–0.075)	0.06 (0.04–0.165)	<0.001
Creatinine, mg/dL	0.99 (0.8–1.22)	1.10 (0.85–1.40)	1.34 (1.07–1.73)	<0.001
Sodium, mEq/L	136.3 ± 3.7	136.6 ± 4.3	136.5 ± 4.7	0.828
Potassium, mEq/L	4.3 ± 0.6	4.4 ± 0.5	4.3 ± 0.7	0.367
Magnesium, mg/dL	2.08 ± 0.31	2.03 ± 0.29	1.94 ± 0.48	0.079
Total cholesterol, mg/dL	170 (138–208)	156 (132–196)	151 (125–175)	0.014
Total protein, g/dL	6.88 ± 0.68	6.83 ± 0.87	6.12 ± 0.69	<0.001
Albumin, g/dL	3.94 ± 0.53	3.68 ± 0.52	3.26 ± 0.49	<0.001
CRP, mg/dL	0.45 ± 0.38	7.41 ± 5.46	65.98 ± 46.01	<0.001
WBC count, cells/µL	8.0 (6.7–9.1)	8.4 (7.3–10.5)	9.1 (7.0–11.0)	0.087
Hemoglobin, g/dL	13.1 ± 2.0	12.4 ± 1.6	11.6 ± 1.9	<0.001
Platelet, 10^3^/µL	242 ± 79	237 ± 77	226 ± 101	0.267
Neutrophil, 10^3^/µL	5.0 ± 2.4	5.9 ± 2.6	7.0 ± 3.0	<0.001
Lymphocyte, 10^3^/µL	2.3 ± 1.6	2.1 ± 1.3	1.4 ± 0.7	<0.001
CAR, ×100	0.1 (0–0.2)	1.6 (0.9–2.6)	18.7 (9.2–28.1)	<0.001
Cardiac devices (ICD or CRT), n (%)	66 (50)	81 (59.6)	95 (69.9)	0.004
Long-term mortality, n (%)	22 (16.7)	50 (36.8)	90 (66.2)	<0.001

Abbreviations: CRF, chronic renal failure; CAD, coronary artery disease; CVD, cerebrovascular disease; AF, atrial fibrillation; SBP, systolic blood pressure; LVEF, left ventricle ejection fraction; ACE-i, angiotensinogen converting enzyme inhibitor; ARB, angiotensinogen receptor blocker; MRA, mineralocorticoid receptor blocker; ASA, acetylsalicylic acid; VKA, vitamin K antagonist; NOAC, novel oral anticoagulant; NT-pro-BNP, N-terminal pro-brain natriuretic peptide; CRP, C-reactive protein; WBC, white blood cell count; CAR, C-reactive protein to albumin ratio; ICD, implantable cardioverter-defibrillator; CRT, cardiac-resynchronization therapy.

**Table 3:** Univariate and multivariate analysis for long-term mortality.

Univariate Analysis			Multivariate Analysis	
	*p* Value	HR (95% CI)	*p* Value	HR (95% CI)
Age (1-year increase)	<0.001	2.750 (2.332–3.168)	0.001	2.008 (1.753–2.309)
CAD	0.010	1.936 (1.046–3.188)	0.087	1.057 (0.903–1.678)
Hypertension	0.002	2.001 (1.462–3.891)	0.056	1.068 (0.958–1.843)
Hyperlipidemia	0.022	1.865 (1.059–3.003)	0.102	1.043 (0.874–1.564)
CRF	0.003	1.996 (1.349–3.447)	0.061	1.081 (0.916–1.981)
AF	0.001	2.205 (1.423–3.418)	0.051	1.071 (0.943–1.780)
LVEF (1% decrease)	0.025	1.431 (1.023–1.891)	0.102	1.018 (0.871–1.291)
Cardiac devices (absence)	<0.001	3.106 (1.488–6.482)	0.001	2.458 (1.592–4.875)
Creatinine (0.1 mg/dL increase)	0.003	1.809 (1.236–2.647)	0.083	1.023 (0.805–1.592)
NT-pro-BNP (50 pg/mL increase)	<0.001	2.987 (1.407–5.003)	0.002	1.967 (1.108–2.709)
Troponin I (0.01 ng/dL increase)	0.042	1.657 (0.963–2.850)	0.456	1.006 (0.786–1.342)
Hemoglobin (1 g/dL decrease)	0.010	0.857 (0.758–0.968)	0.090	1.064 (0.851–1.302)
CAR	<0.001	2.927 (1.617–4.987)	0.005	1.852 (1.124–2.581)

Abbreviations: HR, hazard ratio; CI, confidence interval; CAD, coronary artery disease; CRF, chronic renal failure; AF, atrial fibrillation; LVEF, left ventricle ejection fraction; NT-pro-BNP, N-terminal pro-brain natriuretic peptide; CAR, C-reactive protein to albumin ratio.

## Data Availability

The data described in the manuscript are available upon reasonable request.
